# Oral contraceptives alter oral health

**DOI:** 10.4103/0256-4947.62832

**Published:** 2010

**Authors:** Rajiv Saini, Santosh Saini, Sugandha Sharma

**Affiliations:** a From the Department of Periodontology and Oral Implantology, Rural Dental College, Loni, Maharashtra, India; b From the Department of Microbiology, Rural Dental College, Loni, Maharashtra, India; c From the Department of Prosthodontics, Rural Dental College, Loni, Maharashtra, India

**To the Editor:** The past 50 years have dramatically improved perceptions concerning the actions of sex steroid hormones in health and disease. Basic, clinical, and epidemiological research has not only increased our knowledge of the role of sex steroid hormones in reproductive endocrinology, but has also improved awareness of chronic diseases such as osteoporosis, cardiovascular diseases, cognitive disorders, and periodontal diseases that affect women's health.^1^ In general, women with pre-existing gingival conditions or susceptibility to periodontal disease may experience an exacerbated response to bacterial plaque if they are pregnant, use oral contraceptives, have hormonal replacement therapy, or undergo menopause. Women on hormonal replacement therapy and oral contraceptives experience a statistically significant increase in gingival inflammation.^2^,^3^ With oral contraceptives, this increase in gingival inflammation is related to the duration of use, and results of recent studies suggest that prolonged use of oral contraceptives may detrimentally affect the periodontium.^4^ In the gingiva, numerous clinical studies have characterized sex hormone-induced changes in phenotype, preferential accumulation, and metabolization of estrogen and progesterone, as well as the presence of estrogen and progesterone receptors.^1^ Periodontitis is one of the most ubiquitous diseases^5^ and periodontitis is defined as an inflammatory disease of supporting tissues of teeth caused by specific microorganisms or groups of specific microorganisms, resulting in progressive destruction of the periodontal ligament and alveolar bone with periodontal pocket formation, gingival recession, or both.^6^ Research has demonstrated that the host response to periodontal infection results in the local production of cytokines and biological mediators such as prostaglandins and interleukins, as well as the systemic production of serum antibodies.^5^ Women taking oral contraceptives demonstrate a significant increase in the number of *Prevotella* species in the gingival microflora. Increased female sex hormones substituting for the naphthoquinones required by certain *Prevotella* species most likely are responsible for this rise, and measurable changes have also been observed in the salivary components of women taking sex hormones, including a decrease in concentrations of protein, sialic acid, hexosamine fucose, hydrogen, and total electrolytes; in some studies, changes in salivary flow have been reported.^7^ Women using contraceptives had 16-fold higher levels of *Bacteroides* species^5^ and experience a 2-fold to 3-fold increase in the incidence of localized osteitis following extraction of mandibular third molars.^8^ Thus, because of the effects of hormonal changes and their corresponding impact on the periodontium, proper periodontal evaluation and treatment for these women is recommended. The negative influence of the changes in estrogen and progesterone levels can be controlled by additional plaque control. Current users of oral contraceptives had poorer periodontal health^9^ so dental examination should be an integral part to be followed up strictly and regularly to avoid any future dental complications. Thus, the emergence of periodontal medicine demands that dentists assume a larger accountability for the overall health of their patients, and acquire information of relevant systemic conditions to interrelate more meaningfully with medical colleagues to accomplish the final goal of providing superior patient care.
